# Protective Effects of *Mussaenda pubescens* Leaf Extracts Against Oxidative Stress and Dyslipidemia

**DOI:** 10.1155/bmri/4490125

**Published:** 2026-07-09

**Authors:** Md. Tazim Hasan Fahim, Ummah Tasnim Nisat, Nazratun Noor Maria, Nusrat Jahan, Sajeda Akter, Aftab A. Sorwar

**Affiliations:** ^1^ Department of Pharmacy, University of Science and Technology Chittagong, Chattogram, Bangladesh, ustc.ac.bd; ^2^ Department of Pharmacy, University of Chittagong, Chattogram, Bangladesh, cu.ac.bd; ^3^ Department of Statistics, University of Dhaka, Dhaka, Bangladesh, du.ac.bd; ^4^ Department of Statistics, University of Nebraska–Lincoln, Lincoln, Nebraska, USA, unl.edu

**Keywords:** antihyperlipidemic, cardiovascular disease, *Mussaenda pubescens*, stress

## Abstract

**Objective:**

*Mussaenda pubescens* (Rubiaceae) is a medicinal plant, traditionally utilized for gastrointestinal, febrile, and reproductive ailments. This study was aimed at analyzing the antioxidant capacity and antihyperlipidemic efficacy of the methanolic extract of *M. pubescens* in a high‐cholesterol diet‐induced hyperlipidemic mouse model.

**Methods:**

Twenty‐five Swiss albino mice were randomly subdivided into five groups of five mice each: normal control, disease control, atorvastatin (20 mg/kg), 200 mg/kg of the extract, and 400 mg/kg of the extract. Hyperlipidemia was achieved by feeding a high‐fat diet for 28 days before the oral treatment was given for 12 consecutive days. The serum lipid profile and DPPH radical scavenging activity were then analyzed.

**Results:**

The extract improved the lipid abnormalities when compared with untreated hyperlipidemic mice (*p* < 0.05). At 200 mg/kg, LDL‐C decreased to 11.25 ± 1.05 mg/dL, whereas the 400 mg/kg dose reduced TG and VLDL‐C to 140.50 ± 6.50 and 28.10 ± 1.30 mg/dL, respectively. Atorvastatin demonstrated greater lipid normalization. The antioxidant activity was also shown by the extract with a DPPH assay, with an IC_50_ value of 235.64 *μ*g/mL.

**Conclusion:**

The methanolic extract of *M. pubescens* exhibits potent antihyperlipidemic and antioxidant activities, validating its potential as a promising phytotherapeutic candidate for the management of hyperlipidemia and oxidative stress–associated metabolic disorders. Further in‐depth phytochemical profiling and mechanistic studies are required for better evaluation of this extract.

## 1. Introduction

Cardiovascular disease (CVD) is marked as the primary reason for morbidity and mortality worldwide [1]. The modern diet mostly consists of refined sugars and saturated fats and is deficient in fiber, a major contributor to the rising phenomenon of Type 2 diabetes, metabolic syndrome, insulin resistance, and dyslipidemia [2]. Hyperlipidemia treatment involves exercise, diet control, and the use of lipid‐lowering diets and drugs [3]. The most common drugs used for the treatment of hyperlipidemia are hydroxymethylglutarate coenzyme A (HMG‐CoA) reductase inhibitors, which are also called statins. Bile acid sequestrants, fibrates (e.g., colestipol, clofibrate, and fenofibrate, respectively), cholesterol absorption inhibitors such as ezetimibe, and omega‐3 fatty acids are some of the other drugs used to treat hyperlipidemia [4]. Several drugs are available for hyperlipidemia treatment, but there is still less efficiency and safety for antihyperlipidemic therapy. As an example, there is a severe muscular injury risk with statins, which are efficacious in decreasing LDL [5]. Niacin is a good treatment for reducing triglycerides (TGs), but it may also damage the liver and cause high blood sugar [6]. For this reason, more effective drugs to treat hyperlipidemia still need to be made.

Growing evidence indicates that oxidative stress has a central mechanistic function in the initiation and development of lipid‐driven vascular injury. Reactive oxygen species (ROS) promote lipid peroxidation, LDL oxidation, and endothelial dysfunction, thereby linking dyslipidemia with inflammation and plaque formation [7, 8]. Natural products that combine lipid‐modulatory effects with antioxidant activity are of high translational interest as complementary or alternative therapies for dyslipidemia and CVD prevention [9]. In response, plant‐based therapeutics have gained increasing attention as alternative or adjunct treatments for hyperlipidemia, owing to their favorable safety profiles, bioavailability, and traditional acceptance.

The genus *Mussaenda* (Rubiaceae) is traditionally used in Asian folk medicine for diverse ailments and has attracted scientists′ attention due to the presence of flavonoids, triterpenes, and saponins, classes of secondary metabolites known to possess antioxidant, anti‐inflammatory, and other bioactivities [10–12]. A new study of *Mussaenda pubescens*′s metabolites showed that it contains many different types of phenolic acids, terpenoids, glycosides, and saponins. This supports its chemical promise as a source of antioxidants and bioactive substances [13–15]. *M. pubescens* is utilized for gastrointestinal, febrile, and reproductive ailments, but extensive pharmacological assessment regarding cardiometabolic outcomes, particularly its antihyperlipidemic activity in experimental models, is limited [11, 13]. Here, motivated by the phytochemical richness and traditional use of *M. pubescens*, the hypothesis of the study was to evaluate the antioxidant capacity (DPPH assay) and antihyperlipidemic potential of the methanolic extract of *M. pubescens* in a high‐cholesterol diet‐induced hyperlipidemic Swiss albino mouse model. This investigation is aimed at validating their therapeutic potential and exploring their feasibility as candidates for the alternative therapy of lipid disorders through this in vivo evaluation.

## 2. Materials and Methods

### 2.1. Experimental Plant

*M. pubescens* was gathered from Rangamati, Chattogram Hill Tract, and authenticated (Specimen Nr. SBU 030524‐30) by the botanist Professor Dr. Shaikh Bokhtiar Uddin, Department of Botany, University of Chittagong, which was then preserved by the University of Chittagong herbarium. The collected leaves were powdered to a coarse consistency after proper drying. The powder was preserved in a hermetically sealed container and then soaked in methanol at room temperature for maceration. Maceration was done by submerging about 700 g of plant material in 3.8 L of methanol and stirring from time to time at room temperature for 14 days. After extraction, 25 g of crude methanolic extract (yield 3.57%) was yielded, and it was stored for the next analysis.

### 2.2. Animals of the Experiment

Then, 25–30 g weight male and female adult Swiss albino mice were sourced and separately housed per cage in groups of two under ambient conditions of room temp. (25°C ± 2°C), relative humidity (50% ± 5%), and a 12 h light and dark cycle. They got the standard mice pellets as a diet obtained from the ICDDRB, Dhaka, and water ad libitum [16]. All the animals were adapted to the laboratory surroundings for 1 week before the experiment. The Institutional Ethical Review Committee (IERC) approved the study design, and animal handling was performed according to the rules and regulations of the committee for the study on the animal model with Ethical Clearance Number IERC/USTC/25/010. The analysis followed the ARRIVE guideline [17].

### 2.3. Acute Toxicity

Overnight fasted mice were randomly divided into six groups of six animals each. Mice of two different groups were administered a single dose of 2000 mg/kg body weight of *M. pubescens*. Normal control was given the vehicle alone. The animals were observed individually for the first 1 h for any gross behavioral changes like drowsiness, restlessness, writhing, convulsions, and symptoms of toxicity and mortality, if any, and then periodically for the next 24 h, and then every 24 h for any signs of acute toxicity over a period of 14 days. The acute toxicity study was done as per OECD guidelines in a limit test manner [18].

### 2.4. Phytochemical Screening

A preliminary phytochemical investigation was performed for the *M. pubescens* extract to qualitatively measure the presence of different bioactive compounds, following established protocols [19, 20].

### 2.5. Evaluation of Antioxidant Activity

The free radical scavenging effect of the extracts was evaluated using the DPPH assay. A DPPH solution was prepared by dissolving 0.004 g of DPPH in 100 mL of 99.9% methanol. In each test, 3 mL of DPPH solution was mixed with 1 mL of the test sample in methanol, creating final concentrations of 15.63, 31.25, 62.5, 125, 250, and 500 *μ*g/mL. The mixture was shaken and incubated in the dark at room temperature (25°C–28°C) for 30 min. The color change was measured at 517 nm. Ascorbic acid served as a positive control, and DPPH solution alone acted as a negative control. The percentage inhibition was measured according to the following formula:

%of scavenging effect=1−AsampleAblank×100

## 3. Preparation of High‐Fat Diet

The experimental diet comprises a blend of butter (41%), peanut oil (12%), and a standard laboratory diet (47%). Standard animal food pellets were chopped up using a mortar and pestle and subsequently ground into a fine powder with a mixer grinder. This dehydrated powder was consistently combined with an equal volume of water to form small feed balls, which were then stored in self‐sealing plastic bags in a refrigerator at 2°C–8°C. The control group feed was prepared similarly, utilizing only the standard food pellets, which were ground and mixed with water, excluding the additional ingredients. This feed preparation was conducted for all animals every 3 days [21].

### 3.1. Experiment Protocol

Before starting the study, the mice were adapted for 1 week to the laboratory environment. Following that, 25 mice were randomly distributed into five groups, consisting of five mice in each group.

Group I served as a negative control. All mice were fed a normal diet. Group II was denoted as a positive control, and mice were fed with a high‐fat diet but no treatment. Group III was denoted as standard. This group receives a high‐fat diet and standard atorvastatin drug at a dose of 20 mg/kg body weight. Group IV and Group V were marked as the treatment groups of 200 and 400 mg/kg. Group IV and Group V were fed with a high‐fat diet with extracts. To induce hyperlipidemia, all groups (except the normal control) were given a high‐fat diet for 28 days, and body weight was recorded during this period. After the successful induction, treatment started and continued for 12 consecutive days, at which time the mice were fed either *M. pubescens* extract or the standard drug, in addition to the high‐fat diet. The overall experimental period was therefore 40 days, which consisted of an induction period of 28 days and a treatment period of 12 days. At the end of the experiment, mice were anesthetized with a phenobarbital ip injection, and blood was collected for analysis [22].

### 3.2. Collection of Blood and Organ Weight

The mice were anesthetized with intraperitoneal administration of phenobarbital after 28 days of the experimental phase and 12 days of the treatment period prior to sacrifice. The AVMA permits only the overdose of barbiturates for euthanasia [23, 24]. The room was silent, and the mice were restrained throughout the sacrifice to reduce discomfort. Following the sacrifice, we obtained the blood from the heart and the desired organ in order to determine their weight. After that, the blood was stored in an Eppendorf tube and then placed in an icebox so that it may eventually coagulate. The serum was collected by centrifuging the clotted blood at a speed of 4000 rpm for 10–15 min. The total cholesterol (TC), TGs, low‐density lipoprotein cholesterol (LDL‐C), high‐density lipoprotein cholesterol (HDL‐C), coronary risk index (CRI), and atherogenic index (AI) were measured by commercial diagnostic kits (DIALAB GmbH, Austria). Biochemical estimations were carried out with a semiautomated biochemistry analyzer, according to the manufacturer. We assessed the weight of the mouse organs individually, separating the liver, kidneys, and heart. After that, a statistical comparison was made between the average weight of the liver, kidney, and heart.

## 4. Statistical Analysis

Mean SEM (standard error of the mean) was utilized to express all of the data. Dunnett′s *t*‐test and one‐way ANOVA were used to obtain the *p* value using SPSS software (Version 25.0). Every group was contrasted with the control. *p* < 0.001 was regarded as highly significant, and *p* < 0.05 was regarded as statistically significant.

## 5. Results

### 5.1. Phytochemical Screening

The presence of phytochemicals is mentioned in Table 1.

**Table 1:** Phytochemical screening of methanolic extract of *Mussaenda pubescens*.

Phytochemicals	Test methods	Result
Alkaloids	Mayer′s test	+
	Hager′s test	+
	Dragendroff′s test	+

Flavonoids	Ferric chloride test	+
	Conc. H_2_SO_4_ test	+

Reducing sugar	Benedict′s test	+
	Fehling′s test	+

Carbohydrate	Molisch′s test	+
Glycosides	Aqueous NaOH test	+
Cardiac glycosides	Keller–Killiani test	+
Phenolic compound	Iodine test	+
Tannins	10% NaOH test	+

### 5.2. Acute Toxicity

No apparent adverse effects were observed in the acute toxicity research on mice when doses of the experimental extracts were administered. The mice did not demonstrate symptoms such as decreased motor activity, restlessness, convulsions, loss of consciousness, diarrhea, or increased tear production. Moreover, none of the mice perished throughout the experiment, irrespective of the dosage supplied. As a result, it was discovered that the LD50 of the extracts is higher than 4000 mg/kg, indicating a relatively low degree of acute toxicity at the tested dosages. This shows that the extracts may have a positive safety profile for further inquiry.

### 5.3. Evaluation of Antioxidant Activity

The antioxidant activity (Table 2) of *M. pubescens* was found to be significantly higher than that of ascorbic acid, with values of 235.636 and 270.40 *μ*g/mL, respectively. This suggests that *M. pubescens* possesses strong antioxidant properties, surpassing the standard antioxidant ascorbic acid. The high antioxidant activity may be attributed to the presence of phytochemicals such as flavonoids and tannins, which are known for their ability to scavenge free radicals and protect against oxidative damage.

**Table 2:** Antioxidant activity of ascorbic acid and *Mussaenda pubescens* leaves by using DPPH scavenging assay.

Concentration (*μ*g/mL)	% of scavenging	
	Methanolic extract	Ascorbic acid
500	71.34	85.84
250	57.31	78.36
125	49.39	70.68
62.5	36.89	52.48
31.25	28.04	39.89
15.63	13.1	25.15
IC_50_	235.636	82.774

### 5.4. Organ Weight Analysis

The organ weight data of the hyperlipidemic mice fed the atherogenic diet, and the methanolic extract of *M. pubescens* was summarized in Table 3. However, physiological changes in certain organs, such as the heart and kidney, were observed under the prolonged administration of the high‐fat diet but were not severe enough to be considered significant for the disease control group. The changes were, however, not statistically significant.

**Table 3:** Absolute and relative organ weights of mice in different experimental groups.

	Mean heart weight (g)	Relative weight of liver	Mean liver weight (g)	Relative weight of liver	Mean kidney weight (g)	Relative weight of the kidney
Control	0.182600 ± 0.006	4.72%	1.227500 ± 0.041	4.72%	0.388750 ± 0.01	1.49%
Standard	0.221800 ± 0.037	4.15%	1.483850 ± 0.194	4.15%	0.441600 ± 0.008	1.23%
Positive control	0.166050 ± 0.013	3.77%	1.313200 ± 0.120	3.77%	0.387550 ± 0.003	1.11%
MP ME 200	0.159100 ± 0.003	3.83%	1.374850 ± 0.036	3.83%	0.344550 ± 0.008	1.09%
MP ME 400	0.186600 ± 0.019	3.70%	1.295400 ± 0.159	3.70%	0.353650 ± 0.008	1.26%

### 5.5. Biochemical Examination

The experimental animal′s serum lipid profile is shown in Table 4. Lipid homeostasis is clearly affected by the administration of the high‐fat diet since, compared to the negative control group, the disease control group showed an increase in serum TC, TGs, LDL‐C, and VLDL‐C. These observations proved the induction of hyperlipidemia was successful.

**Table 4:** Effect of *Mussaenda pubescens* in high‐fat diet‐induced Swiss albino mice.

Group	Cholesterol (mg/dL)	TG (mg/dL)	HDL (mg/dL)	LDL (mg/dL)	VLDL (mg/dL)
Negative control	145.00 ± 20.00	150.00 ± 2	90.40 ± 44.60	47.60 ± 2.00	30.00 ± 0.40
Disease control	223.00 ± 11.00	245.50 ± 8.50	132.50 ± 4.50	60.75 ± 5.45	49.10 ± 1.70
Standard	111.75 ± 18.75	90.00 ± 2^a^ ^∗^^b^ ^∗∗∗^	70.00 ± 21.00	15.35 ± 2.95^a^ ^∗^^b^ ^∗∗∗^	18.00 ± 0.40^a^ ^∗∗∗^^b^ ^∗∗∗^
MP ME 200	138.70 ± 24.40	161.00 ± 10.00^b^ ^∗∗∗^^c^ ^∗∗∗^	92.50 ± 25.50	11.25 ± 1.05^a^ ^∗∗^^b^ ^∗∗∗^	32.20 ± 2.00^a^ ^∗∗∗^^b^ ^∗∗∗^^c^ ^∗∗∗^
MP ME 400	154.45 ± 41.45	140.50 ± 6.50^b^ ^∗∗∗^^c^ ^∗∗∗^	120.00 ± 1.00	14.20 ± 2.10^a^ ^∗∗^^b^ ^∗∗∗^	28.10 ± 1.30^a^ ^∗∗^^b^ ^∗∗∗^^c^ ^∗∗∗^

*Note:* Data are expressed as mean ± SEM (n = 6). Statistical analysis was performed using one‐way ANOVA followed by Tukey′s multiple comparison test.

Abbreviation: MP ME, *Mussaenda pubescens* methanolic extract.

^a^Values compared with the negative control group, receiving the vehicle (normal food).

^b^Values compared with the disease control group, disease control receiving only the high‐fat feed without any dose.

^c^Values compared with the standard (atorvastatin) treatment group.

∗*p* < 0.05.

∗∗*p* < 0.01.

∗∗∗*p* < 0.001.

The disease control group exhibited the highest levels of TC (223.00 ± 11.00 mg/dL), TG (245.50 ± 8.50 mg/dL), LDL‐C (60.75 ± 5.45 mg/dL), and VLDL‐C (49.10 ± 1.70 mg/dL), reflecting severe dyslipidemic changes induced by the high‐fat diet. Interestingly, atorvastatin is seen to have a very strong effect on all lipid parameters, bringing TG, LDL‐C, and VLDL‐C down to 90.00 ± 2.00, 15.35 ± 2.95, and 18.00 ± 0.40 mg/dL, respectively.

*M. pubescens* extract was found to have significant antihyperlipidemic properties, as well. The extract also significantly lowered LDL‐C by 11.25 ± 1.05 mg/dL when administered at 200 mg/kg, compared with several cases of the atorvastatin‐treated group, indicating that a high dose level of the extract has a strong LDL‐lowering effect. This decrease was less pronounced than that induced by atorvastatin at 200 mg/kg; however, there was a decrease in TG and VLDL‐C at 200 mg/kg.

The dose of 400 mg/kg further reduced the levels of TG and VLDL‐C, which were reduced to 140.50 ± 6.50 and 28.10 ± 1.30 mg/dL, respectively, and suggest higher TG‐lowering activity at the higher dose. However, the LDL‐C reduction was slightly lower at 400 mg/kg (14.20 ± 2.10 mg/dL) compared to the dose of 200 mg/kg. Therefore, there was a nonlinear dose effect observed; the lower dose had a more pronounced effect against LDL‐C levels, with more moderate effects against the reduction of TG and VLDL‐C levels, while the higher dose had more moderate effects against the reduction of TG and VLDL‐C levels and more pronounced effects against the reduction of LDL‐C levels compared to the lower dose.

In general, treatment with atorvastatin showed the best effect over the overall lipid normalization, but the effect of *M. pubescens* extract was comparable in selected lipid parameters, such as reduction in LDL‐C, suggesting that the extract of *M. pubescens* can be used as a natural antihyperlipidemic agent.

#### 5.5.1. AI

Table 5 shows the effect of the methanolic extract of *M. pubescens* on the AI. The AI value was higher in the disease control group than in the negative control group, suggesting high CVD risk in a high‐fat diet (0.27).

**Table 5:** Atherogenic index of *Mussaenda pubescens* methanolic extract.

Atherogenic index	Log TG/HDL		
	TG	HDL	AI
Negative control	150.00	90.40	0.22
Positive control	245.50	132.50	0.27
Standard	90.00	70.00	0.11
MP ME 200	161.00	92.50	0.24
MP ME 400	140.50	120.00	0.07

Abbreviation: MP ME, *Mussaenda pubescens* methanolic extract.

The addition of statins to the diet had a significant effect on lowering the AI value to 0.11, a strong protection against diet‐induced atherogenic changes. The AI values of both doses were also improved following *M. pubescens* treatment. The AI value of the 200 mg/kg treated group was moderately decreased compared to the untreated group, whereas the 400 mg/kg treated group had the lowest AI value (0.07), lower than that of atorvastatin treatment.

Based on the results obtained, it has been concluded that there is a dose‐dependent improvement in AI after the administration of the extract, and the higher dose shows better cardio protection. The greater the decrease in AI at 400 mg/kg, the better the TG/HDL cholesterol balance, indicating decreased susceptibility to atherosclerotic risk.

The decrease in the level of AI after extract treatment implies the improvement in lipid profile balance, which also implies reducing the atherogenic risk, which concludes that *M. pubescens* is cardioprotective against dyslipidemia brought about by a diet.

#### 5.5.2. CRI

The CRI values for the experimental groups are given in Table 6. The coronary risk because of hyperlipidemia was higher in the disease control group with a high‐fat diet feeding (1.68) than in the negative control group (1.60).

**Table 6:** Coronary risk index of *Mussaenda pubescens* methanolic extract.

Coronary risk index	TC/HDL		
	TC	HDL	CRI
Negative control	145.00	90.40	1.60
Positive control	223.00	132.50	1.68
Standard	111.75	70.00	1.60
MP ME 200	138.70	92.50	1.49
MP ME 400	154.45	120.00	1.29

Abbreviation: MP ME, *Mussaenda pubescens* methanolic extract.

Atorvastatin reduced the CRI value to 1.60, restoring the CRI close to the level of the normal control. Treatment with the extract of *M. pubescens* yielded additional doses itself as a reduction in CRI values. The 200 mg/kg dose compounded the CRI to be 1.49, and the 400 mg/kg compound gave the best improvement (CR 1.29).

*M. pubescens* extract had better reduction in CRI at higher doses when compared with atorvastatin, indicating a more potent cardioprotective effect at higher doses. The gradual drop in CRI values to higher doses of extract further demonstrates its efficacy in promoting improvements in cardiovascular risk parameters associated with lipids in hyperlipidemic mice.

## 6. Discussion

Hyperlipidemia is a major yet manageable risk factor for CVDs worldwide, playing a key role in the development of atherosclerosis and heart attacks. Phytochemical‐rich medicinal plants have been shown to have antihyperlipidemic and antioxidant properties. A 2023 study supported the therapeutic efficacy of plant‐derived bioactive substances in the management of dyslipidemia by showing that polyphenol‐rich herbal formulations effectively lowered circulating LDL‐C and residual cholesterol levels in high‐cholesterol patients [25]. A 2024 study on *Ficus religiosa* indicated substantial decreases in serum lipid levels in hyperlipidemic rats caused by a high‐fat diet, which correlated this effect to flavonoids and phenolic compounds that can modulate HMG‐CoA reductase activity [26]. Methanolic extract of *M. pubescens* exhibited significant antihyperlipidemic and antioxidant activity in mice fed with a high‐fat diet in the present study. The extract had a beneficial effect on lipid homeostasis under the experimental hyperlipidemic condition, as it led to a reduction in elevated levels of serum TGs, LDL‐C, and VLDL‐C in comparison to the disease control group.

In the untreated animals with the control disease, the high‐fat diet proved to be effective by significantly increasing the levels of serum TC, TG, LDL‐C, and VLDL‐C. These were all some of the parameters that were improved with *M. pubescens* treatment, especially with LDL‐C and TGs. Nevertheless, while atorvastatin demonstrated superior overall lipid‐normalizing efficacy, the plant extract also produced measurable lipid‐lowering effects—an observation consistent with the traditional attribution of lipid‐metabolic modulation to this species and one that underscores the potential contribution of its phytochemical constituents to this process. Although established pharmacological agents remain the standard of care for dyslipidemia management, naturally derived compounds continue to garner considerable scientific interest as candidate complementary therapeutics.

The two doses tested did not lead to a perfectly linear response of lipid parameters. The reductions in LDL‐C were slightly greater for 200 mg/kg, and the reductions in TG, VLDL‐C, the AI, and the CRI were comparatively greater for 400 mg/kg. This nonlinear dose–response relationship is commonly observed in phytochemical‐rich extracts, but it was not the intent of the current study to identify the mechanism behind this observation [25, 26]. Therefore, explanations involving biphasic pharmacological responses, feedback regulation, or differential phytochemical interactions remain speculative and were not experimentally validated in the present study. The positive changes seen in both AI and CRI after extract treatment also reinforce the possibility of protecting against cardiovascular risk induced by the diet. These indices are important parameters of atherogenicity associated with dyslipidemia, and a reduction in these parameters indicates overall improvement in lipid balance in treated animals. However, these results are preliminary and should be interpreted in the context of the current preclinical model.

Based on the preliminary phytochemical screening, flavonoids, phenolic compounds, tannins, glycosides, and alkaloids were detected in the sample, which have been known to exhibit antioxidant and lipid‐modulating properties in medicinal plants. This DPPH assay has also shown an antioxidant activity for the extract, evidencing a scavenging effect against free radicals. The antihyperlipidemic effects observed may be partly related to antioxidant‐mediated mechanisms that decrease the oxidative stress due to lipid abnormalities. Like this, other pathways like HMG‐CoA reductase inhibitors, activation of LDL receptor activity, or modulation of PPAR‐*α*, which were not directly tackled in the present study, are also plausible and might be investigated as potential areas of interest for future studies.

No statistically significant changes were found in the liver, kidney, and heart weights in the experimental groups, implying negligible gross morphological changes occurring in these organs during the treatment period as a consequence of the extract. However, the weight of organs alone cannot be used to prove a lack of toxicity. Further investigations, such as determination of hepatic and renal enzyme levels in serum, histopathology, and chronically repeated toxicology testing, are required to make conclusive assessments of safety.

In general, the results suggest antioxidant and antihyperlipidemic effects in the experimental animal model of *M. pubescens*. Future study should focus on bioassay‐guided fractionation and extraction of lipid‐lowering and antioxidant active ingredients, followed by thorough structure–activity relationship studies. In vitro HMG‐CoA reductase inhibition and LDL receptor expression experiments might assist in identifying the molecular mechanisms. Histopathological investigation, comprehensive metabolic panels, and verified toxicity endpoints must be performed in long‐term in vivo studies before clinical application. Moreover, pharmacokinetic profiling of the extract and its primary elements would reveal bioavailability, metabolism, and elimination. The DPPH assay showed promising antioxidant activity; thus, future research should examine the extract′s effects on oxidative stress biomarkers like malondialdehyde and superoxide dismutase in vivo to better understand antioxidant‐mediated lipid regulation. Finally, standardized, phytochemically defined formulations and human clinical trials are needed to prove *M. pubescens*′ therapeutic potential as a natural alternative to atorvastatin.

## 7. Conclusion

In the present work, the methanolic extract of *M. pubescens* demonstrated in vitro antioxidant potential and antihyperlipidemic activity in mice fed a high‐fat diet model. The use of the extract was associated with improvement in some lipid parameters, such as TGs, LDL‐C, VLDL‐C, AI, and CRI, and the DPPH assay demonstrated free radical scavenging activity. The results indicate that the plant could possess bioactive molecules with regulating ability on lipid metabolism and oxidative stress under experimental conditions.

Even though these positive trends were noted, there are some critical limitations to the study. The number of animals sampled was relatively small, the treatment length was short, and mechanistic pathways were not experimentally investigated. Further, only organ weight analysis was used for safety assessment, usually without any detailed biochemical or histopathological assessment of toxicity. Thus, one has to be mindful of both safety and efficacy before coming to any conclusions.

The phytochemicals present (flavonoids and phenolic compounds) could partially explain the actions, but the pathways within the cells (HMG‐CoA reductase inhibition or the activation of PPAR‐*α*) are hypothetical and would need further experimental verification. Thus, detailed phytochemical characterization, dose‐optimization studies, pharmacokinetic evaluation, mechanistic validation, chronic toxicity evaluation, and clinical translation should be studied in the future in light of the above findings.

To conclude, *M. pubescens* is a promising plant source for bioactive compounds with antihyperlipidemic and antioxidant activity, and continued detailed and rigorous preclinical studies are needed to fully prove the potential of such compounds for therapy for these conditions and others where they might be used.

## 8. Description

Using a model of high‐fat diet‐induced hyperlipidemia in Swiss albino mice, this study assessed the antioxidant and antihyperlipidemic properties of *M. pubescens* extract. The effects on serum lipid profile were evaluated after blood samples were taken from animals that had been treated with plant extract at doses of 200 and 400 mg/kg. By decreasing TC, TGs, and LDL levels while increasing HDL, the extract markedly ameliorated dyslipidemia, suggesting beneficial regulation of lipid metabolism. The existence of bioactive components was found using phytochemical screening, and the DPPH assay showed a significant ability to scavenge free radicals, indicating that there may be an antioxidant mechanism that helps protect the cardiovascular system. For the treatment of hyperlipidemia and the avoidance of cardiovascular risk, the results point to *M. pubescens* as a possible natural remedy candidate.

## Author Contributions

M.T.H.F.: data curation, formal analysis, funding acquisition, investigation, methodology, software, and writing—original draft. U.T.N.: conceptualization, data curation, formal analysis, funding acquisition, investigation, methodology, project administration, resources, software, supervision, validation, visualization, writing—original draft, and writing—review and editing. N.N.M.: data curation, formal analysis, investigation, methodology, software, validation, visualization, writing—original draft, and writing—review and editing. N.J.: formal analysis, methodology, software, supervision, visualization, and writing—review and editing. S.A.: formal analysis, software, and writing—original draft. A.A.S.: resources and software. Md. Tazim Hasan Fahim and Ummah Tasnim Nisat contributed equally to this article.

## Funding

No funding was received for this manuscript.

## Conflicts of Interest

The authors declare no conflicts of interest.

## Data Availability

The data that support the findings of this study are available from the corresponding author upon reasonable request.
